# Changes of biochemical biomarkers in the serum of children with convulsion status epilepticus: a prospective study

**DOI:** 10.1186/s12883-022-02686-2

**Published:** 2022-05-27

**Authors:** Manli Wang, Jian Yu, Xiao Xiao, Bingbing Zhang, Jihong Tang

**Affiliations:** 1 Department of Neurology, Children’s Hospital of Soochow University, No.92, Zhongnanjie Road, Suzhou, 215025 Jiangsu China; 2 Department of Neonatology, Children’s Hospital of Soochow University, Suzhou, 215025 Jiangsu China

**Keywords:** Convulsion status epilepticus, Serum biochemical biomarkers, Viral encephalitis, Primary generalized epilepsy, Complex febrile seizures

## Abstract

**Background:**

Convulsive status epilepticus (CSE) is a common neurologic emergency with high morbidity and mortality. This single center study is aimed to assess changes of serum biochemical biomarkers after seizure, and their associations with the development of CSE.

**Methods:**

From January 2015 to October 2016, a total of 57 CSE patients, and 30 healthy controls without central nervous system diseases were enrolled in Children’s Hospital of Soochow University. CSE patients were further divided into viral encephalitis (VEN, 13 cases), primary generalized epilepsy (PGE, 30 cases), and complex febrile seizures (CFS, 14 cases). The levels of serum biochemical biomarkers were measured using the corresponding commercial ELISA kits. Logistic regression analysis was performed to identify the associations between these biomarkers and diseases.

**Results:**

At the 1^st^ and 4^th^ day of CSE, neuron-specific enolase (NSE; 1^st^ day: 20.553 ± 5.360, 4^th^ day: 10.094 ± 3.426) and vascular endothelial growth factor (VEGF; 1^st^ day: 153.504 ± 31.246, 4^th^ day: 138.536 ± 25.221) in the CSE group were increased compared to the control (NSE: 6.138 ± 1.941; VEGF: 119.210 ± 31.681). Both the levels of S-100 calcium binding protein B (S-100B; 1^st^ day: 0.738 ± 0.391) and C-reactive protein (CRP; 1^st^ day: 11.128 ± 12.066) were elevated at 1^st^ day of CSE (S-100B: 0.387 ± 0.040; CRP: 3.915 ± 2.064). For glial fibrillary acidic protein (GFAP), it was remarkably upregulated at 4^th^ day of CSE (3.998 ± 1.211). NSE (*P* = 0.000), S-100B (*P* = 0.000), CRP (*P* = 0.011), and VEGF (*P* = 0.000) at 1^st^ day of CSE, and NSE (*P* = 0.000), VEGF (*P* = 0.005), and GFAP (*P* = 0.000) at 4^th^ day of CSE were significantly associated with the occurrence of CSE. Besides, NSE (*P* = 0.002), S-100B (*P* = 0.001), and VEGF (*P* = 0.002) at 4^th^ day of CSE were significantly associated with VEN.

**Conclusions:**

The levels of serum NSE, S-100B, CRP, VEGF, and GFAP are associated with CSE**.**

## Background

Status epilepticus (SE) is generally categorized as convulsive status epilepticus (CSE) and non-convulsive status epilepticus (NCSE) [1]. CSE, the main subtype of SE, is a common neurologic emergency with high morbidity and mortality in children each year [2, 3]. It not only causes cerebral ischemia/hypoxia, brain edema, and neuron damage, but also results in cardiac arrests, secondary infection, and internal environment disorders [4]. It is noticed that there were approximately 20–40% of CSE patients with a high risk for secondary brain dysfunction [5], which is manifested as behavioral abnormality, emotional disorder, language retardation, and learning ability decline, or even the serious ones have chronic persistent sequelae, with restricted limb activity, intellectual disorder and cerebral palsy [6].

Lowenstein et al. have shed light on an operational and a conceptual definition of CSE, which is defined as … ≥ 5 min of (i) continuous seizure or (ii) two or more discrete seizures between which there is incomplete recovery of consciousness [7]. In addition, as a basic research (or conceptual) definition of CSE, the International League Against Epilepsy Core Group on Classification group further suggested the following: CSE refers to a condition in which there is failure of the “normal” factors that serve to terminate a typical generalized tonic–clonic seizures [8]. Based on the definition of CSE, the optimal treatment time is within 5 min after seizure and it is likely that a seizure will continue if not treated after this time [9]. If the time is prolonged to 30 min, there may be a risk of long-term consequences [10]. Therefore, accurate early diagnosis and correct treatments are the keys to the successful rescue. Generally, viral encephalitis (VEN), primary generalized epilepsy (PGE), and complex febrile seizures (CFS) are the three main causes of CSE [1, 11, 12] The degrees of brain injury caused by different causes of CSE are different [1, 11, 12]. Recent years, increasing attentions have been paid to the potential efficacies of relevant specific biochemical biomarkers for the diagnosis and prognosis of CSE or related diseases [13–19]. For instance, Knopman et al. found that the concentration of serum neuron-specific enolase (NSE) in temporal lobe epilepsy (TLE) patients with SE was higher than that of controls [13]. Both Chang et al. and Steinhoff et al. indicated that relatively high level of S-100 calcium binding protein B (S-100B) is determined in TLE patients with higher frequencies of seizures [14, 15]. Madžar et al. have demonstrated that C-reactive protein (CRP) serves as a biomarker in the inflammatory processes of SE and high CRP concentration is strongly associated with in-hospital mortality and poor prognosis [16]. Similarly, hyperneuroinflammation emerging in SE may also increase the level of glial fibrillary acidic protein (GFAP) in mice [17]. In addition, Cho et al. and Nicoletti et al. have revealed that the elevated expression of vascular endothelial growth factor (VEGF) plays a neuroprotective role in SE murine model [18, 19]. Early detection of relevant specific biochemical biomarkers may have guiding significance for diagnosis and prognosis of CSE. Although the researches on the biochemical biomarkers of CSE are not rare, they are mostly the basic research on animal experiments or adults. The common induction factors of SE in children may be different from those in animals and adults, and the significances of these biochemical biomarkers may be also different [20]. Therefore, the measurements for serum biochemical biomarkers in CSE children may be helpful to judge the disease condition and estimate the prognosis.

In this study, the levels of NSE, S-100B, CRP, VEGF, and GFAP in serum of CSE children were explored. In addition, their associations with CSE were also analyzed in logistic regression models.

## Methods

### Patients and controls

**Fig. 1:**
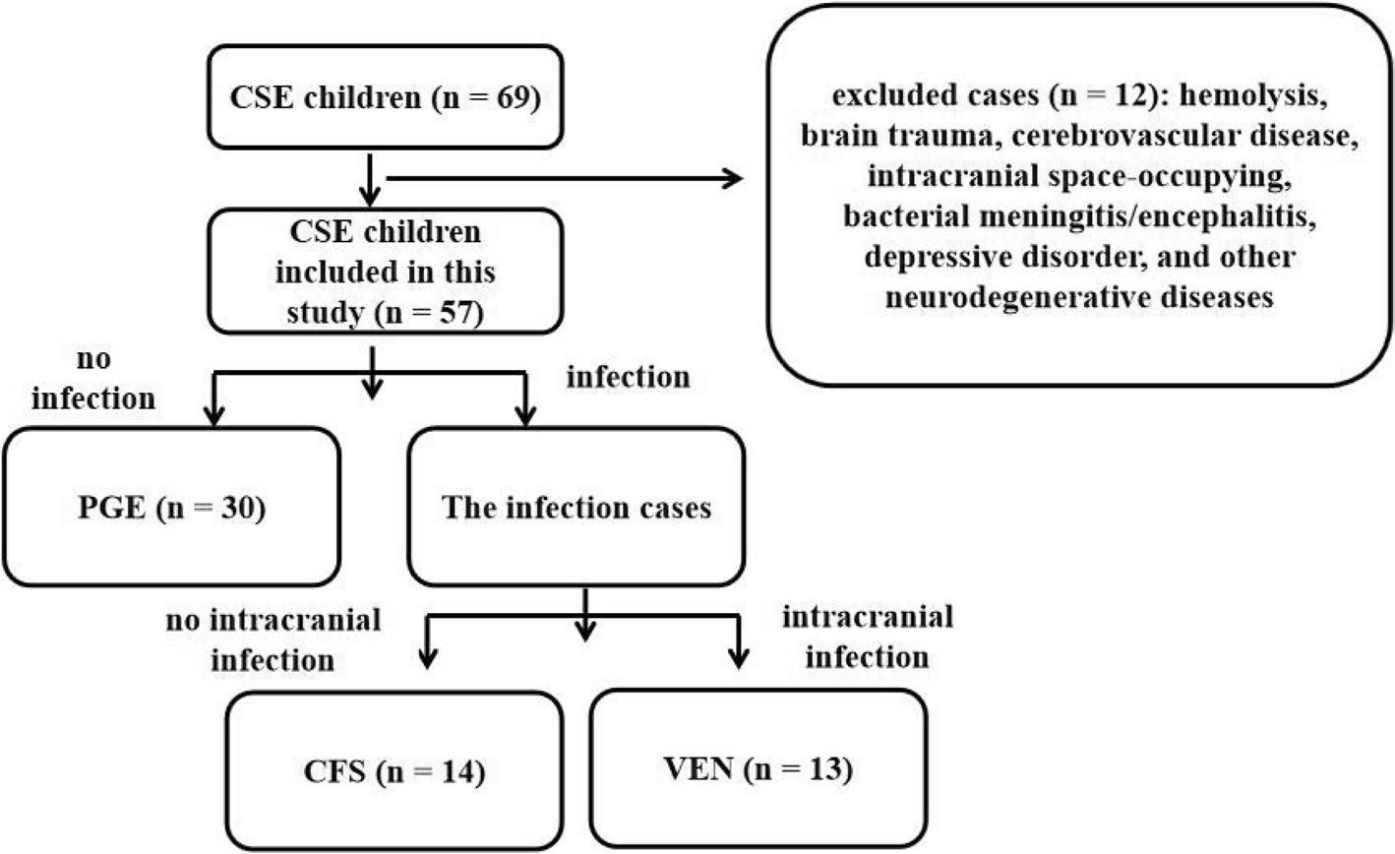
A flow chart presenting included and excluded patients. CSE, convulsive status epilepticus; PGE, primary generalized epilepsy; CFS, complex febrile seizures; VEN, viral encephalitis

Based on the most recent definition of CSE [8], a total of 57 patients (age range: from 1 to 133 months; females: 25, males: 32) with CSE were enrolled in Children’s Hospital of Soochow University from January 2015 to October 2016. All the patients underwent magnetic resonance imaging (MRI) examination. The exclusion criteria were as follows: patients with other diseases affecting serum biochemical biomarkers within 24 h after the onset, such as hemolysis, brain trauma, cerebrovascular disease, intracranial space-occupying, bacterial meningitis/encephalitis, depressive disorder, and other neurodegenerative diseases. The included children were further divided into viral encephalitis (VEN, 13 cases), primary generalized epilepsy (PGE, 30 cases), and complex febrile seizures (CFS, 14 cases) (Fig. 1). In addition, 30 healthy children without central nervous system diseases were served as the controls (age range: from 1 to 9 years old; females: 15, males: 15). In accordance with the Declaration of Helsinki, written informed consent was provided by each patient’s legal guardian (usually the next of kin). This single center study obtained the approval of the ethics committee in our hospital (approval ID: 2019KS005). We collected these clinical data for further study.

### Samples collection

Venous blood samples (3 mL) from healthy controls or CSE patients (at 1^st^, 4^th^, and 10^th^ day of CSE, respectively) were collected using an evacuated tube system into a citrate-anticoagulant tube. All the blood samples were submitted for examination in time to exclude hemolysis. Then, blood samples were centrifuged (2000 r/min) for 10 min to separate the serum and stored in − 80 °C for further experiments.

### Analysis for serum biochemical biomarkers

According to the manufacturer’s protocol, the levels of NSE, S-100B, CRP, VEGF, and GFAP in serum were analyzed using the corresponding commercial ELISA kits (Elabscience Biotechnology Co., Ltd, Wuhan, China).

### Statistical analysis

SPSS 20.0 software (Chicago, USA) was used for statistical analysis. One-way ANOVA followed by Tukey's multiple comparisons test (homogeneity of variance) and Kruscal-Wallis test followed by Dunn’s multiple comparisons test (heterogeneity of variance) were used to assess the experimental data of Tables 2 and 4. Binary logistic regression analysis was performed to identify the associations between serum biochemical biomarkers and CSE in Table 3, and the associations between serum biochemical biomarkers and VEN, an induction factor of CSE. Data were shown as means ± SD. *P*-value less than 0.05 indicated a statistically significant difference.

## Results

### Analysis for the seizure time under different induction factors of CSE

Of all cases, the range of seizure time was from 30 min to 3 weeks. As shown in Table 1, among these cases with the seizure time for more than 24 h, 4 cases were in the VEN group, which accounts for 30.8% (4/13) of the cases in this group, suggesting that the seizure time induced by acute intracranial infection was longer. In addition, there were 3 cases in the PGE group (3/30; 10%) and no cases in the CFS (0/14; 0%) group. The results implied that the seizure duration of CFS patients was shorter.

**Table 1:** The seizure time under different induction factors of CSE

	VEN (*n* = 13)	PGE (*n* = 30)	CFS (*n* = 14)
Gender (male/female)	8/5	10/20	10/4
Age (year)			
< 1	7	12	1
1–5	3	17	13
> 5	3	1	0
Seizure time (h)			
< 1	5	17	12
1–24	4	10	2
> 24	4	3	0

*VEN* viral encephalitis, *PGE* primary generalized epilepsy, *CFS* complex febrile seizures

### Analysis for the levels of serum biochemical biomarkers in children with CSE at different phases

We then analyzed the the levels of NSE, S-100B, CRP, VEGF, and GFAP in CSE children at different phases (at 1^st^, 4^th^, and 10^th^ day of CSE). As shown in Table 2, NSE and VEGF in the CSE group were significantly higher than the control group at 1^st^ day (*P* < 0.01) and 4^th^ day (*P* < 0.05). The levels were decreased at 10^th^ day, which was not statistically different compared to the control group. S-100B was significantly higher in the CSE group than the control group at 1^st^ day (*P* < 0.0 1), and was gradually reduced at 4^th^ day. At 10^th^ day, it returned to the level of the control group. CRP is also an index elevated in the early stage of CSE. We noticed that the level of CRP was significantly increased compared with the control group at 1^st^ day (*P* < 0.01), whereas it was decreased sharply at 4^th^ and 10^th^ day. In terms of GFAP, its level was gradually elevated at 1^st^ day and reached to the highest value at 4^th^ day of CSE, in which was statistically different compared to the control group (*P* < 0.01). At 10^th^ day of CSE, although there was no significant difference for GFAP level between the GSE group and control group, GFAP in serum of CSE children still stayed at a relatively high level.

**Table 2:** Changes of serum biochemical biomarkers in children with CSE at different phases

Group	N	NSE (μg/l)	S-100B (μg/l)	CRP (mg/l)	VEGF (ng/l)	GFAP (ng/l)
Control median	30	6.138 ± 1.941 6.395	0.387 ± 0.040 0.395	3.915 ± 2.064 4.055	119.210 ± 31.681 117.66	2.471 ± 1.272 2.28
1 day median	57	20.553 ± 5.360^**^ 21.198	0.738 ± 0.391^**^ 0.583	11.128 ± 12.066^*^ 7.15	153.504 ± 31.246^**^ 153.28	2.826 ± 1.124 2.68
4 day median	57	10.094 ± 3.426^**##^ 10.433	0.468 ± 0.180^##^ 0.398	3.608 ± 2.261^##^ 3.310	138.536 ± 25.221^*^ 136.53	3.998 ± 1.211^**##^ 3.562
10 day median	57	6.90 ± 1.961^##&&^ 6.845	0.361 ± 0.073^##&&^ 0.375	3.568 ± 2.607^##^ 3.000	120.646 ± 44.21^##&^ 109.43	2.916 ± 0.777^&&^ 2.844

^*^P < 0.05, **P < 0.01 vs. the control group; #*P* < 0.05, ##*P* < 0.01 vs. the 1 day group; &*P* < 0.05, &&*P* < 0.01 vs. the 4 day group. *NSE* neuron-specific enolase, *VEGF* vascular endothelial growth factor, *S-100B* S-100 calcium binding protein B, *CRP* C-reactive protein, *GFAP* glial fibrillary acidic protein

### Association between the levels of serum biochemical biomarkers in children with CSE at different phases and the occurrence of CSE

On logistic regression analysis (Table 3), NSE (*P* = 0.000, 95% CI: 1.093–1.272), S-100B (*P* = 0.000, 95% CI: 1.029–1.072), CRP (*P* = 0.011, 95% CI: 1.000–1.018), and VEGF (*P* = 0.000, 95% CI: 1.010–1.029) at 1^st^ day of CSE, and NSE (*P* = 0.000, 95% CI: 1.309–2.071), VEGF (*P* = 0.005, 95% CI: 1.008–1.046), and GFAP (*P* = 0.000, 95% CI: 1.038–6.651) at 4^th^ day of CSE were all significantly and independently associated with the occurrence of CSE. However, we also found that the five serum biochemical biomarkers are not associated with the occurrence of CSE at 10^th^ day.

**Table 3:** Associations with the occurrence of CSE in binary logistic regression analysis

Seizure (day)	Biomarkers	B	*P*	Exp (B)	95% CI for Exp (B)
1	NSE	0.165	0.000	1.179	1.093–1.272
	S-100B	0.049	0.000	1.050	1.029–1.072
	CRP	0.010	0.011	1.010	1.000–1.018
	VEGF	0.019	0.000	1.020	1.010–1.029
	GFAP	0.007	0.098	1.007	0.999–1.015
4	NSE	0.499	0.000	1.647	1.309–2.071
	S-100B	0.010	0.287	1.010	0.992–1.028
	CRP	-0.065	0.531	0.937	0.765–1.148
	VEGF	0.026	0.005	1.027	1.008–1.046
	GFAP	1.289	0.000	1.979	1.038–6.651
10	NSE	0.204	0.092	1.266	0.967–1.554
	S-100B	-8.462	0.060	0.000	0.000–1.440
	CRP	-0.059	0.524	0.943	0.786–1.130
	VEGF	0.001	0.869	1.001	0.990–1.013
	GFAP	0.568	0.051	1.766	0.997–3.126

*NSE* neuron-specific enolase, *VEGF* vascular endothelial growth factor, *S-100B* S-100 calcium binding protein B, *CRP* C-reactive protein, *GFAP* glial fibrillary acidic protein

### Analysis for the levels of serum biochemical biomarkers in children with CSE under the three etiological factors

Afterwards, the levels of NSE, S-100B, CRP, VEGF, and GFAP in CSE children in the VEN, PGE, and CFS groups were assessed. The results for Table 4 were as follows:

**Table 4:** Changes of serum biochemical biomarkers in children with CSE under the three etiological factors

Biomarkers	Seizure (day)	VEN (*n* = 13)	PGE (*n* = 30)	CFS (*n* = 14)
NSE	1	22.213 ± 4.663	21.152 ± 4.824	17.727 ± 6.29
(μg/l)	4	13.333 ± 3.107	9.228 ± 2.852^**^	8.943 ± 3.140^**^
	10	7.521 ± 1.765	6.637 ± 1.902	6.880 ± 2.252
S-100B	1	1.417 ± 0.151	0.528 ± 0.112^**^	0.554 ± 0.112^**^
(μg/l)	4	0.744 ± 0.173	0.398 ± 0.074^**^	0.363 ± 0.384^**^
	10	0.388 ± 0.051	0.360 ± 0.080	0.333 ± 0.066
CRP	1	14.727 ± 14.007	11.554 ± 13.448	6.871 ± 3.110
(mg/l)	4	3.668 ± 2.045	4.079 ± 2.364	2.547 ± 1.997
	10	4.917 ± 3.392	3.378 ± 2.285	2.722 ± 2.080
VEGF	1	168.428 ± 42.316	144.693 ± 27.884	158.529 ± 19.728
(ng/l)	4	159.401 ± 25.578	132.933 ± 21.607^*^	131.167 ± 22.990^*^
	10	117.093 ± 27.350	121.951 ± 58.054	121.146 ± 13.974
GFAP	1	2.751 ± 0.895	2.891 ± 1.348	2.756 ± 0.789
(ng/l)	4	3.693 ± 1.634	4.232 ± 1.189	3.782 ± 0.657
	10	2.892 ± 0.661	3.001 ± 0.912	2.755 ± 0.541

^*^P < 0.05, **P < 0.01 vs. the VEN group. *NSE* neuron-specific enolase, *VEGF* vascular endothelial growth factor, *S-100B* S-100 calcium binding protein B, *CRP* C-reactive protein, *GFAP* glial fibrillary acidic protein, *VEN* viral encephalitis, *PGE* primary generalized epilepsy, *CFS* complex febrile seizures

**NSE:** NSE in the VEN (22.213 ± 4.663), PGE (21.152 ± 4.824), and CFS (17.727 ± 6.29) groups all reached to a relatively high level at 1^st^ day of CSE with no significant differences. It was decreased in the above three groups at 4^th^ day of CSE. Interestingly, the level of NSE in both PGE (9.228 ± 2.852) and CFS (8.943 ± 3.140) groups was lower than that in the VEN group (13.333 ± 3.107) (*P* < 0.01). From this time point to the 10^th^ day of CSE, NSE was gradually decreased with no significant differences among the three groups.

**VEGF:** Similar patterns were obtained in the analysis result of VEGF. Also decreased level of VEGF was observed in the PGE (132.933 ± 21.607) and CFS (131.167 ± 22.990) groups compared with the VEN group (159.401 ± 25.578) (*P* < 0.01) at 4^th^ day of CSE.

**CRP:** CRP level was increased sharply in all three groups at 1^st^ day of CSE, decreased at 4^th^ day, and reached to a stable level at 10^th^ day. There were no significant differences among the three groups throughout the development of CSE.

**GFAP:** From 1 to 10 day, the level of GFAP in the three groups was increased firstly and then decrease. Similar to the results of CRP, there were also no significant differences among the three groups throughout the development of CSE.

**S-100B:** At 1^st^ day of CSE, S-100B in the PGE (0.528 ± 0.112) and CFS (0.554 ± 0.112) was remarkably reduced by contrast to that of the VEN group (1.417 ± 0.151) (*P* < 0.01). Although S-100B level was decreased in all three groups after seizure for 4 day, significant differences were also exhibited between the VEN group (0.744 ± 0.173) and the PGE (0.398 ± 0.074)/CFS (0.363 ± 0.384) group (*P* < 0.01). At 10^th^ day of CSE, there were no significant differences among the three groups.

In addition, as presented in Table 5, NSE (*P* = 0.002, 95% CI: 1.228–2.577), S-100B (*P* = 0.001, 95% CI: 1.091–1.415), and VEGF (*P* = 0.002, 95% CI: 1.018–1.084) after seizure for 4 day, and CRP (*P* = 0.044, 95% CI: 1.006–1.629) at 10^th^ day of CSE were significantly and independently associated with VEN.

**Table 5:** Associations with VEN in binary logistic regression analysis

Seizure (day)	Biomarkers	B	*P*	Exp (B)	95% CI for Exp (B)
1	NSE	0.095	0.140	1.100	0.969–1.249
	S-100B	85.312	0.996	1.123E + 037	0.000-
	CRP	0.029	0.233	1.029	0.982–1.079
	VEGF	0.020	0.061	1.020	0.999–1.042
	GFAP	-0.082	0.783	0.921	0.513–1.653
4	NSE	0.576	0.002	1.779	1.228–2.577
	S-100B	0.217	0.001	1.242	1.091–1.415
	CRP	0.016	0.911	1.016	0.771–1.337
	VEGF	0.049	0.002	1.051	1.018–1.084
	GFAP	-0.317	0.303	0.728	0.398–1.332
10	NSE	0.216	0.195	1.241	0.895–1.721
	S-100B	0.026	0.189	1.027	0.987–1.068
	CRP	0.247	0.044	1.280	1.006–1.629
	VEGF	-0.003	0.744	0.997	0.979–1.015
	GFAP	-0.053	0.900	0.948	0.415–2.166

*NSE* neuron-specific enolase, *VEGF* vascular endothelial growth factor, *S-100B* S-100 calcium binding protein B, *CRP* C-reactive protein, *GFAP* glial fibrillary acidic protein

## Discussion

SE is traditionally defined as any seizure lasting longer than 30 min, whether or not consciousness is impaired or seizures recur without an intervening period of consciousness [21]. It is reported that CSE becomes increasingly resistant to anticonvulsant drugs with the extension of seizure time, it can be associated with extremely high morbidity and mortality if not treated promptly and aggressively [22]. In the current study, we mainly explored the levels of NSE, S-100B, CRP, VEGF, and GFAP in CSE children as well as the associations with CSE to develop a new diagnosis and treatment method for children CSE.

Numerous studies are focused on the possible role of relevant specific biochemical biomarkers during the period of seizure [13–19], which can be the potential biomarkers for further researches and future diagnosis. NSE is located in the mature neurons and neuroendocrine cells of the nervous system with the highest content in the brain and it will enter cerebrospinal fluid and blood when blood–brain barrier is destroyed and permeability increases [23]. Approximately 96% of S-100B protein is existed in the brain, which level reflects the injury and severity of astrocytes [24]. CSE is generally accompanied with the occurrence of inflammation, and CRP is one of the most sensitive biomarkers for inflammation [16]. VEGF is a specific mitogen of endothelial cells, which is involved in the neural processes such as growth, differentiation, survival, regeneration, and neuroprotection [25]. GFAP is an important cytoskeletal component and a specific marker of astrocytes [26]. Summarily, the concentrations of these biochemical biomarkers in blood can reflect the severity of brain injury. In this study, we initially explored the associations between biochemical biomarkers levels and the occurrence of CSE. Interestingly, we demonstrated that the levels of NSE and VEGF were significantly elevated in serum of children at 1^st^ and 4^th^ day of CSE compared to that of controls, which was consistent with the previous results [13, 18, 19]. In addition, we further found sharply decreased NSE and VEGF after seizure for 10 day. Logistic regression analysis also confirmed the levels of NSE and VEGF at 1^st^ and 4^th^ day of CSE may be risk factors for increased seizure frequency. For the data of NSE, DeGiorgio et al. uncovered the similar results, which indicated that serum NSE level for the 19 subjects within 24 h after SE was significantly elevated compared with the levels for normal and epileptic controls [27]. In addition, they also demonstrated that serum NSE level was highest in those with an acute neurologic insult, and the level of serum NSE was also significantly elevated compared with control levels in 11 patients without acute neurologic insults other than SE [27]. Therefore, they concluded that in the absence of an acute insult or lesion, NSE was elevated as a direct result of SE [27]. Based on this previous experiment data, we believed that NSE may be also an effective specific biomarker of CSE. Additionally, the results further implied that VEGF may have no diagnostic values in advanced CSE (10 day later). S-100B level was also increased at 1^st^ day of CSE, which was strongly associated with CSE. Interestingly, a recent study on epilepsy seizure of TLE patients conducted by Chang et al. indicated a relatively high level of S-100B in TLE patients with higher frequencies of seizures [14]. Meanwhile, Gunawan et al. also revealed that there is an increase in serum S-100B level within 24 h after seizure in patients with SE, and this has a strong positive correlation with brain damage seen in head MRI and diffusion tensor imaging (DTI) [28]. We speculated that S-100B may be used as a more sensitive biochemical marker of brain injury in early stage of CSE, especially within one day. Similar to the results of S-100B, CRP is also a biomarker elevated in the early stage of CSE (at 1^st^ day of CSE). Given that CRP is a sensitive marker in the inflammatory processes of SE [16], we speculated the early stage of anti-inflammatory therapies may have significance in the treatment of CSE to some extent. However, Sutter et al. have voiced concern that single level of CRP is not reliable for diagnosis of infections during SE [29] and not independently associated with refractory epileptic activity and death [30]. The data implied that further studies are needed to assess the potential of CRP for inclusion in prediction models allowing to identify patients with poor outcome of SE. Combined our results obtained above, we further considered using linear changes of CRP level to assess its role in CSE in future studies. In addition, Wang et al. reported that hyperneuroinflammation leads to an increase of GFAP level in a SE mouse model [17]. Similarly, a higher level of GFAP in CSE children than control was found in this study, and the time point was at 4^th^ day of CSE. At the same time, the level of GFAP at 4^th^ day of CSE was also determined a risk factor in the occurrence of CSE using a logistic regression analysis. Interestingly, Gurnett et al. found that the level of cerebrospinal fluid (CSF) GFAP is increased in SE children, and the increased CSF GFAP is strongly correlated with seizure duration [31]. Evidence has demonstrated that biomarkers are firstly released into the CSF compartment prior to release into serum when SE occurs [32]. Furthermore, Saija et al. believed that the elevation in the serum compartment that occurs after SE may reflect the increase in permeability of the blood–brain barrier that is known to occur after SE. We speculated that the high level of serum GFAP may be used as an effective biomarker to reflect the brain injury after CSE.

Additionally, we further investigated the associations between these biochemical biomarkers and VEN/PGE/CFS using logistic regression models. We found that NSE level in VEN children was relatively high than that of PGE children or CFS children. Also strong association between NSE level at 4^th^ day of CSE and VEN was determined by logistic regression analysis. Our results were consistent with one of mechanisms for the increased NSE level in CSE children: direct invasion of neurons by pathogens leads to edema, necrosis and myelin breakdown of neurons, destruction of blood brain barrier, and release of NSE in cytoplasm into cerebrospinal fluid and blood [33]. At the same time, increased S-100B at 1^st^ and 4^th^ day of CSE was observed in VEN children compared to the PGE children or CFS children, which was in line with the previous results [33]. However, further logistic regression analysis demonstrated the level of S-100B at 4^th^ day of CSE was a risk factor for VEN. Similar to the data of the above two biomarkers, VEGF level was upregulated at 4^th^ day of CSE. Interestingly, a recent study on VEN children also reported that the serum VEGF level in severe VEN was higher than that in normal control group and mild VEN group [34]. Combined the results of the above three biomarkers, we further speculated that the brain injury of patients with central nervous system infection such as VEN within one week after seizure is more serious than that of PGE and CFS.

Additionally, there are also some limitations of this study such as a less varied population in which research cases limited to CSE caused by VEN, PGE and CFS, so the results cannot be generalized to other disorders. Meanwhile, the relatively small sample size may also affect the accuracy of the acquired data. In addition, the most important limitation of this study is the fact that VEN was not fully excluded and is a major confounder, as encephalitis can increase the biomarkers and lead to more severe CSE and poor outcomes.

## Conclusions

In a word, in the early stage of CSE (within one week), the increased levels of NSE, S-100B, and VEGF may serve as effective diagnostic markers for CSE, and relatively high level of CRP represents hyperneuroinflammation. Meanwhile, GFAP can be used as an effective biomarker to reflect the brain injury after CSE. In addition, we indicated that the increased levels of NSE, S-100B, and VEGF may be risk factors for VEN, which is considered as one of the main induction factors of CSE.

## Data Availability

The data that support the findings of this study are available from Children’s Hospital of Soochow University, but restrictions apply to the availability of these data, which were used under license for the current study, and so are not publicly available. Data are however available from the corresponding author (Jihong Tang) upon reasonable request and with permission of Children’s Hospital of Soochow University.

## Abbreviations

CSE: Convulsive status epilepticus
SE: Status epilepticus
NCSE: Non-convulsive status epilepticus
VEN: Viral encephalitis
PGE: Primary generalized epilepsy
EEG: Electroencephalography
NSE: Neuron-specific enolase
TLE: Temporal lobe epilepsy
CRP: C-reactive protein
GFAP: Glial fibrillary acidic protein
VEGF: Vascular endothelial growth factor
